# Astilbin prevents bone loss in ovariectomized mice through the inhibition of RANKL‐induced osteoclastogenesis

**DOI:** 10.1111/jcmm.14713

**Published:** 2019-10-11

**Authors:** Haiming Jin, Qingqing Wang, Kai Chen, Ke Xu, Hao Pan, Feifan Chu, Zhen Ye, Ziyi Wang, Jennifer Tickner, Heng Qiu, Chao Wang, Jacob Kenny, Huazi Xu, Te Wang, Jiake Xu

**Affiliations:** ^1^ Key Laboratory of Orthopaedics of Zhejiang Province The Second Affiliated Hospital and Yuying Children’s Hospital of Wenzhou Medical University Wenzhou Zhejiang China; ^2^ School of Biomedical Sciences The University of Western Australia Perth WA Australia

**Keywords:** Astilbin, bone resorption, osteoclast, osteoclastogenesis, osteoporosis, RANKL

## Abstract

Osteoporosis is the most common osteolytic disease characterized by excessive osteoclast formation and resultant bone loss, which afflicts millions of patients around the world. Astilbin, a traditional herb, is known to have anti‐inflammatory, antioxidant and antihepatic properties, but its role in osteoporosis treatment has not yet been confirmed. In our study, astilbin was found to have an inhibitory effect on the RANKL‐induced formation and function of OCs in a dose‐dependent manner without cytotoxicity. These effects were attributed to its ability to suppress the activity of two transcription factors (NFATc1 and c‐Fos) indispensable for osteoclast formation, followed by inhibition of the expression of bone resorption‐related genes and proteins (Acp5/TRAcP, CTSK, V‐ATPase‐d2 and integrin β3). Furthermore, we examined the underlying mechanisms and found that astilbin repressed osteoclastogenesis by blocking Ca^2+^ oscillations and the NF‐κB and MAPK pathways. In addition, the therapeutic effect of MA on preventing bone loss in vivo was further confirmed in an ovariectomized mouse model. Therefore, considering its ability to inhibit RANKL‐mediated osteoclastogenesis and the underlying mechanisms, astilbin might be a potential candidate for treating osteolytic bone diseases.

## 1 INTRODUCTION

Bone is continuously remodelled inside the body. In the adult human body, nearly 10% of bone tissue is replaced every year.^1^ Maintenance of the bony skeleton is highly dependent on dynamic coordination between osteoblasts and osteoclasts (OCs).^2^ However, excessive bone resorption by OCs relative to bone formation by osteoblasts disequilibrates the balance between OCs and osteoblasts, which leads to numerous pathological abnormalities of the bony skeleton.^3^ Pathological changes in OCs, including abnormalities in number and activity, may be a major cause of osteopenic diseases, such as osteolytic lesions and osteoporosis.^4^ Therefore, the treatment of osteoporosis is highly dependent on inhibiting the formation and function of OCs.

OCs originate from haematopoietic stem cells.^5^ After the stimulation caused by two main regulatory factors, namely macrophage colony‐stimulating factor (M‐CSF) and receptor activator of nuclear factor kappa B (NF‐κB) ligand (RANKL), OCs eventually form from monocytes/macrophages and fuse to generate multinucleated cells.^6^ The activation of monocytes/macrophages and the subsequent survival and proliferation of preosteoclasts or mature OCs rely on the binding between M‐CSF and colony‐stimulating factor‐1 receptor (cFMS).^7^ RANKL plays an essential role in the differentiation of OC precursor cells. Once RANKL binds with receptor activator of nuclear factor kappa B (RANK) on the membrane of a preosteoclast, adaptor molecules such as TNF receptor‐associated factor (TRAF6) will be recruited to stimulate NF‐κB, mitogen‐activated protein kinase (MAPK) and calcium signalling pathways for OC differentiation and formation.^8^, ^9^ Then, the two main transcription factors in OC differentiation, activator protein 1 (AP‐1) and nuclear factor of activated T cells, cytoplasmic 1 (NFATc1), will be triggered to stimulate preosteoclast maturation and improve the levels of OC function‐related gene and protein expression, including tartrate resistant acid phosphatase (TRAcP/*acp5*), cathepsin K (CTSK), vacuolar‐type H^+^‐ATPase d2 (V‐ATPase d2) and integrin beta 3 (integrin β3).^8^, ^10^, ^11^ Based on these facts, drugs that inhibit RANKL‐induced osteoclastogenesis may possess great therapeutic value for treating osteoporosis.

Astilbin, which was first isolated from the rhizome of *Astilbe thunbergii*, is a natural flavonoid compound that has been widely used for its various pharmacological properties.^12^ Previous studies have reported that astilbin exerts various bioactivities, including antihepatic,^13^ antioxidant,^14^ antidiabetic nephropathy,^15^ anti‐inflammatory^16^ and antiarthritic properties. Furthermore, it has also been reported that astilbin can affect the NF‐κB signalling pathway by inhibiting myeloid differentiation factor 88 (MyD88), p65 and inhibitor kappa B kinase β (IKKβ) for the treatment of chronic inflammatory disorders such as rheumatoid arthritis.^17^ In addition, astilbin has been shown to have no genotoxicity potential, which further indicates the great value of this compound for clinical applications.^18^ However, the efficiency of applying astilbin for curing osteoporosis, and the mechanism by which astilbin inhibits OCs have not yet been investigated. As the mechanism of RANKL‐induced osteoclastogenesis is critically regulated by the NF‐κB signalling pathway, we proposed that astilbin may also function in suppressing the formation of OCs.

In this study, we characterized the role of astilbin in RANKL‐induced osteoclastogenesis and its underlying mechanism in vitro. We demonstrated that astilbin not only inhibited the formation of OCs but also decreased the bone resorption activity of OCs by affecting the NFATc1, NF‐κB and MAPK signalling pathways. Furthermore, our results showed the protective effect of astilbin against bone loss in an oestrogen deficiency‐induced osteoporosis mouse model. Thus, our data indicate that astilbin may have promising effects against osteolytic diseases, indicating its potential value in the treatment of osteoporosis.

## 2 MATERIALS AND METHODS

### 2.1 Cell culture

Primary bone marrow monocytes (BMMs) were extracted from the femoral bone marrow of C57BL/6 mice at 6 weeks of age using procedures approved by the Animal Ethics Committee of the University of Western Australia (RA/3/100/1244). BMMs were cultured in modified minimal essential medium, alpha modification (α‐MEM; Gibco‐Invitrogen), supplemented with 10% (v/v) foetal bovine serum (FBS; Thermo Fisher Scientific), 1% (v/v) penicillin/streptomycin (Gibco‐Invitrogen) and 50 ng/mL M‐CSF (produced according to a previously described protocol^19^). RAW264.7 cells were obtained from the American Type Culture Collection (Manassas, VA) and cultured in complete α‐MEM, as described above.

### 2.2 Drug screening assay and osteoclastogenesis assay

BMMs at passage two were used to screen for drugs that effectively suppressed RANKL‐induced osteoclastogenesis. The cells were separated into 96‐well plates at a density of 5 × 10^3^ cells per well and incubated in the presence of M‐CSF (50 ng/mL) overnight for adherence. Then, the cells were stimulated with 50 ng/mL glutathione S‐transferase (GST)‐rat RANKL (rRANKL, produced according to a protocol described in a previous article 20) and 50 ng/mL M‐CSF. In addition, natural compounds at 10 µmol/L (for the screening assay) or varying concentrations of astilbin were added. In the control (ctrl) group, cells were treated without RANKL and astilbin. The complete medium was changed every two days until OCs formed on the sixth day. To observe the status of OC formation, the cells were fixed with 2.5% glutaraldehyde for 15 minutes and then stained for TRAcP enzymatic activity. TRAcP‐positive multinucleated cells that had more than three nuclei were counted as OCs.

### 2.3 Cytotoxicity assays

BMMs were seeded into 96‐well plates at a density of 5 × 10^3^ cells per well and incubated overnight until the cells were confluent. Then, the cells were treated with increasing concentrations of astilbin (1, 2.5, 5, and 10 μmol/L) for 48 hours. Next, a 3‐(4,5‐dimethylthiazol‐2‐yl)‐5‐(3‐carboxymethoxyphenyl)‐2‐(4‐sulfophenyl)‐2H‐tetrazolium (MTS) solution (20 μL/well, Thermo Fisher Scientific) was added to each well and incubated with the BMMs for an additional 2 hours. The absorbance was read at 490 nm by a microplate reader (Multiscan Spectrum; Thermo Labsystems). The results were calculated using GraphPad Prism version 5.0.

### 2.4 Immunofluorescence staining and confocal microscopy

BMMs were seeded at a density of 5 × 10^3^ cells per well with M‐CSF (50 ng/mL) overnight. The cells were then treated with M‐CSF (50 ng/mL) and GST‐rRANKL (50 ng/mL) for six days to form mature OCs, with or without varying concentrations (5 and 10 μmol/L) of astilbin. Then, the cells were fixed with 4% paraformaldehyde, permeabilized with 0.1% Triton X‐100 in phosphate‐buffered saline (PBS) and blocked with 3% bovine serum albumin (BSA) in PBS. The cells were then incubated with rhodamine‐conjugated phalloidin (Thermo Fisher Scientific) for 45 minutes in the dark to stain F‐actin. The cells were washed with PBS and stained with 4',6‐diamidine‐2'‐phenylindole dihydrochloride (DAPI), and images were captured on a confocal fluorescence microscope (Nikon; A1 PLUS) at 100× magnification.

### 2.5 Hydroxyapatite resorption assay

A hydroxyapatite resorption assay was used to measure the function of the induced OCs. BMMs were first seeded in a 6‐well collagen‐coated plate (BD Biosciences, Australia) with 1 × 10^5^ cells in each well and then stimulated with 50 ng/mL M‐CSF and 50 ng/mL GST‐rRANKL every 2 days to promote OC formation. Then, 1 mL of Non‐enzymatic Cell Dissociation Solution (Sigma‐Aldrich, Australia) was added to each well to dissociate the cells from the collagen plate, and equal numbers of cells were transferred to the wells of a hydroxyapatite‐coated plate (CLS3989, Corning, NY). Mature OCs were cultured in complete medium containing GST‐rRANKL (50 ng/mL) and M‐CSF (50 ng/mL) in the presence or absence of astilbin (5 μmol/L and 10 μmol/L). After 2 days, the wells were separated into two groups. One group was used to count the number of multinucleated cells in each well by TRAcP staining, as described above. The other group was used to measure the resorbed areas by bleaching for 10 minutes and removing the cells from the wells. Images of the resorbed areas were captured by microscopy and analysed by ImageJ software (NIH, Bethesda, MD) to determine the percentage of the area that was resorbed by the OCs.

### 2.6 Luciferase reporter assays

RAW 264.7 cells were stably transfected with two types of luciferase reporter constructs, p‐NF‐κB‐TA‐Luc and p‐NFAT‐TA‐Luc, which respond to NF‐κB and NFATc1, respectively, as previously described.^21^, ^22^, ^23^ The transfected cells were then seeded into 48‐well plates at a density of 1.5 × 10^4^ cells/well and allowed to adhere overnight and subsequently pre‐treated with varying concentrations of astilbin (1, 2.5, 5 and 10 μmol/L) for 1 hour. Afterwards, the cells were stimulated by GST‐rRANKL (50 ng/mL) for 6 hours (NF‐κB measurement) or 24 hours (NFAT measurement). The cells were then harvested for luciferase activity analysis using a BMG Polar Star Optima luminescence reader (BMG Labtech), as previously described.^24^

### 2.7 Quantitative RT‐PCR analysis

BMMs were plated in 6‐well plates at 1 × 10^5^/well, with or without various concentrations of astilbin in the presence of M‐CSF (50 ng/mL) and GST‐rRANKL (50 ng/mL) for 5 days. TRIzol (Qiagen, Hilden, Germany) was used to extract total RNA from the cells according to the manufacturer's protocol. The experimental procedures were performed strictly as described previously.^25^ Using Moloney murine leukaemia virus reverse transcriptase with an oligo‐dT primer, single‐stranded cDNA was reverse‐transcribed from 2 µg of total RNA. The resulting cDNA was then used for real‐time PCR based on SYBRGreen (Imgenex, Littleton, CO, USA) with the specific primers displayed in Table 1. The expression levels were normalized to Hprt (reference gene) expression. The fold change was determined using the Livak equation, and ratios were calculated relative to the vehicle group.

**Table 1:** Primer sequences used in qRT‐PCR

Genes	Forward (5′‐>3′)	Reverse (5′‐>3′)	Tm (°C)
Nfatc1	GGAGAGTCCGAGAATCGAGAT	TTGCAGCTAGGAAGTACGTCT	60
C‐fos	GCGAGCAACTGAGAAGAC	TTGAAACCCGAGAACATC	60
Acp5	TGTGGCCATCTTTATGCT	GTCATTTCTTTGGGGCTT	59
Ctsk	GGGAGAAAAACCTGAAGC	ATTCTGGGGACTCAGAGC	60
Hprt	GTTGGGCTTACCTCACTGCT	TAATCACGACGCTGGGACTG	60

### 2.8 Western blotting

To examine the expression of bone resorption‐related proteins or components of the NFATc1 signalling pathway, BMMs were seeded (1 × 10^5^ cells/well) into 6‐well plates and incubated with or without astilbin (10 μmol/L) in the presence of M‐CSF (50 ng/mL) and GST‐rRANKL (50 ng/mL) for 5 days. The cells were then lysed, and total protein was harvested using radioimmunoprecipitation assay (RIPA) lysis buffer (containing 100 g/mL phenylmethylsulfonyl fluoride (PMSF), 500 g/mL DNase I and phosphatase inhibitors) for the following periods: 0, 1, 3 and 5 days. For short‐term signalling pathway analysis, BMMs were seeded (2 × 10^5^ cells/well) into 6‐well plates and incubated in complete medium with M‐CSF overnight. The next day, the cells were incubated with serum‐free medium for 4 hours and then pre‐treated with astilbin for 2 hours. Then, the cells were stimulated with GST‐rRANKL (50 ng/mL), and total protein was harvested by RIPA lysis at the following time‐points: 0, 10, 20, 30 and 60 minutes. The proteins were loaded and separated by 10% sodium dodecyl sulphate‐polyacrylamide gel electrophoresis (SDS‐PAGE). Next, the separated proteins were transferred onto nitrocellulose membranes (Whatman) and blocked in 5% skim milk for 1 hour. The membranes were incubated overnight at 4°C with the following primary antibodies: anti‐NFATc1 (1:1000; Santa Cruz Biotechnology), anti‐c‐Fos (1:2000; Santa Cruz Biotechnology), anti‐integrin β3 (1:1000; Santa Cruz Biotechnology), anti‐CTSK (1:2000, Santa Cruz Biotechnology), anti‐V‐ATPase‐d2 (1:1000; Santa Cruz Biotechnology), anti‐IκB‐α (1:1000; Santa Cruz Biotechnology), anti‐p‐ERK1/2 (1:1000, Santa Cruz Biotechnology), anti‐ERK1/2 (1:1000; Santa Cruz Biotechnology), anti‐p‐JNK1/2 (1:1000; Cell Signaling Technologies), anti‐JNK1/2 (1:5000, 1:1000; Cell Signaling Technologies), anti‐p‐p38 (1:1000; Cell Signaling Technologies), anti‐p38 (1:1000; Cell Signaling Technologies) and anti‐β‐actin (1:3000; Cell Signaling Technologies); later, the membranes were immersed for 1 hour in corresponding horseradish peroxidase‐conjugated secondary antibodies. Finally, the membranes were treated with enhanced chemiluminescence (ECL) reagents (Amersham, USA) according to the manufacturer's instructions. The images were visualized using an Image quant LAS 4000 system (GE Healthcare).

### 2.9 Ca^2+^ oscillation measurement

BMMs (1.5 × 10^4^) were seeded in 48‐well plates, with added components for different groups. In the treatment group, the cells were treated with GST‐rRANKL (50 ng/mL), M‐CSF (50 ng/mL) and astilbin (10 μmol/L); in the positive control group, cells were treated with GST‐rRANKL (50 ng/mL) and M‐CSF (50 ng/mL) but were not exposed to astilbin; and in the control group, the cells were treated with M‐CSF alone (50 ng/mL). After cultured for 24 hours, the cells were washed twice with assay buffer (Hank's balanced salt solution with 1 mmol/L probenecid and 2% foetal calf serum (FCS)) and stained with Fluo4 staining solution (Fluo4‐AM dissolved in 20% (w/v) pluronic‐F127 in dimethyl sulfoxide (DMSO) added to assay buffer) in the dark at 37°C for 45 minutes. When staining was completed, the cells were rinsed again with assay buffer and incubated on a bench in the dark for 20 minutes after removing the staining solution. The intensity of fluorescence was observed under fluorescent light (at an excitation wavelength of 488 nm) by an inverted fluorescence microscope (Nikon, Tokyo, Japan). Images were captured every 2 seconds for 4 minutes. Cells with at least two oscillations were counted as oscillating cells. The average amplitude of each oscillating cell was analysed by Nikon Basic Research Software as previously described.^22^

### 2.10 Mouse ovariectomy procedures

Female C57BL/6 mice (10 weeks, n = 30) were purchased from the Animal Center of the Chinese Academy of Science (Shanghai, China) and randomly divided into three equal groups: a sham group, an ovariectomized (OVX) group and an OVX + astilbin (10 mg/kg) group. After one week of adjustable feeding, an ovariectomy operation based on a previously described method was performed for the OVX group and the OVX + astilbin group, whereas a sham operation was performed for the sham group as a control. Seven days after surgery, astilbin (10 mg/kg) was intraperitoneally injected into the mice in the OVX + astilbin group, and PBS was intraperitoneally injected into the mice in the sham and OVX groups. The concentrations of astilbin in vivo studies were determined according the results in vitro, references and the clinical application of this compound.^26^, ^27^ The injections were continued every two days for a total of 6 weeks. Then, the mice were killed, and the femurs were removed for micro‐CT (μCT) analysis as previously described.^22^, ^25^

### 2.11 µCT scanning

Right femur samples were analysed by a SkyScan 1176 µCT instrument (SkyScan; Bruker). Images were acquired using a 50‐kV X‐ray tube voltage, a 500‐μA current, an isotropic pixel size of 9 μmol/L (1600 × 2672‐pixel image matrix) and a 0.5‐mm‐thick aluminium filter for beam hardening. The images were reconstructed using NRecon Reconstruction software (Bruker micro‐CT). A refined volume of 0.5 mm below the growth plate and 1 mm in height was then chosen for further qualitative and quantitative analysis using DATAVIEWER and CTVox software (Bruker micro‐CT). Data, including the bone volume/tissue volume ratio (BV/TV), trabecular thickness (Tb.Th), trabecular number (Tb.N) and trabecular separation (Tb.Sp), were analysed by CTAn software (Bruker micro‐CT, Kontich, Belgium) as described previously.^25^

### 2.12 Histological and histomorphometric analysis

The right femurs samples were fixed in 4% paraformaldehyde for 24hour and then were decalcified in 14% ethylenediaminetetraacetic (EDTA, PH = 7.4) in 37 ℃ for 7 days. After decalcification, the femurs were embedded in paraffin and cut in the sagittal plane to produce approximately 5‐μm‐thick sections. TRAcP staining was performed by using TRAcP staining kits as described previously.^25^, ^28^ The number of claret‐red granules in the vicinity of the resorbed bone was counted as TRAcP‐positive cells. The osteoclast number and the percentage of osteoclasts per bone surface (OcS/BS, %) were calculated according to a method proposed by Sawyer et al.^29^

### 2.13 Statistical analysis

Most values were obtained from three or more independent experiments performed in triplicate. Data are presented as means ± standard deviation (SD). The significance of differences between results was determined by Student's *t* test and ANOVA with multiple testing corrections. A *P* value of <.05 was considered statistically significant.

## 3 RESULTS

### 3.1 Astilbin suppresses RANKL‐induced OC differentiation

**Figure 1:**
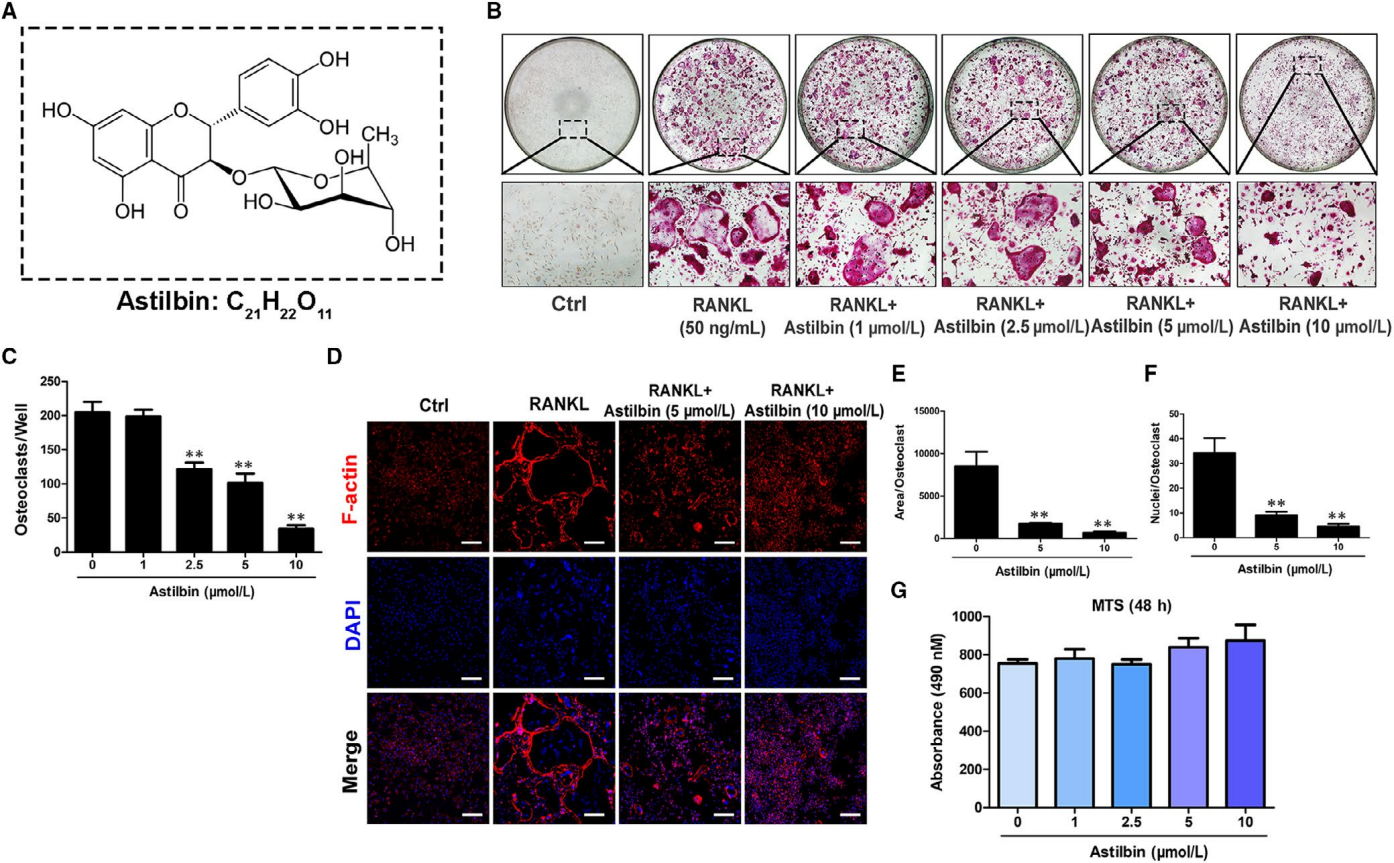
Astilbin suppresses RANKL‐induced OC differentiation in a dose‐dependent manner. A, Chemical structure of astilbin. B, Representative images of OCs after treatment with astilbin at increasing concentrations (magnification = ×100). C, The number of TRAcP^+^ multinucleated cells (nuclei > 3) per well (96‐well plate) was quantitatively analysed. (D) Untreated OCs and OCs treated with 5 μmol/L and 10 μmol/L astilbin were stained and visualized for F‐actin and nuclei. Scale bar = 200 μm. E, F, Quantification of the number of OCs per area and the mean number of nuclei in each cell. G, The effects of the indicated concentrations of astilbin on BMMs were measured by an MTS assay. Data are expressed as means ± SD; **P < .01 relative to the control group

**Figure 2:**
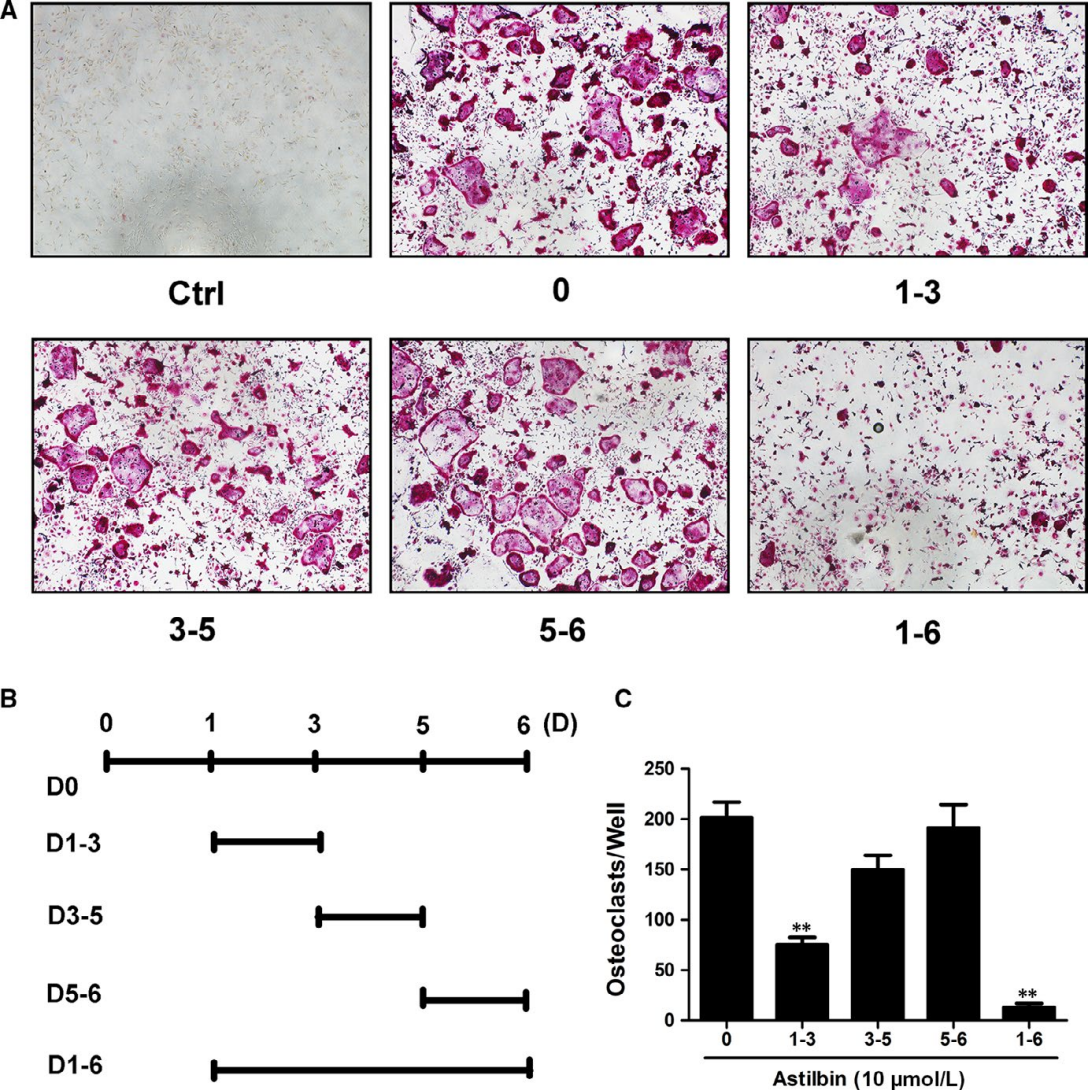
Astilbin suppresses RANKL‐induced osteoclastogenesis in the early stages. A, B, Representative images of TRAcP^+^ cells under treatment with 10 µmol/L astilbin on set days (magnification = ×4). C, TRAcP‐stained cells (nuclei > 3) treated with astilbin over different periods were quantitatively analysed for OC formation (n = 3). Data are presented as means ± SD; **P < .01 relative to RANKL‐induced controls

Numerous natural compounds at a concentration of 10 μmol/L were added into the osteoclastogenesis assay as candidates to screen their inhibitory function in the RANKL‐induced formation of OCs from BMMs (Table 2). Among those compounds, astilbin was found to significantly inhibit OC formation, as presented in Table 2 and Figure 1A. To examine whether the suppressive effect of astilbin is dose‐dependent, increasing concentrations of astilbin, varying from 1 to 10 μmol/L, were added to BMMs pre‐treated with RANKL and M‐CSF. After 5 days of treatment, the cells were then stained with TRAcP buffer to visualize the formation of OCs. As depicted in Figure 1B and Figure S1, the number of TRAcP‐positive cells significantly decreased in a dose‐dependent manner in each well when the concentration of astilbin was >2.5 μmol/L. OC nuclear fusion is also an important step in the formation of mature OCs. To further investigate the effect of astilbin on OC mergence, rhodamine‐phalloidin and DAPI stainings were used to observe the sum of nuclei per OC. The results demonstrated that after treating with astilbin at concentrations of 5 and 10 μmol/L, both the average area of each OC and the average number of nuclei in each OC were dramatically reduced (Figure 1D‐F and Figure S2). These results were consistent with the results obtained from TRAcP staining. To examine whether the inhibitory effect of astilbin on OC formation was due to cell cytotoxicity, an MTS assay was performed to measure the cell viability of BMMs after treatment with increasing concentrations of astilbin. Figure 1G shows that relative to the control group, astilbin did not decrease the number of BMMs, which proved that the attenuating effect of astilbin on OC generation from BMMs was not caused by cytotoxicity. Based on this result, we then characterized the time course of the effect of astilbin on OC differentiation. As shown in Figure 2A‐C, BMMs were treated with astilbin for several different periods (1‐3, 3‐5, 5‐6 and 1‐6 days). TRAcP‐positive cells were significantly decreased when astilbin was present on days 1‐3 and 1‐6, whereas the effects weakened when astilbin was present on days 3‐5 or 5‐6 (Figure 2B), indicating that astilbin plays an early role in inhibiting OC differentiation. In conclusion, these results suggest that astilbin inhibits RANKL‐induced osteoclastogenesis by abrogating OC differentiation in the early stage but does not cause cell cytotoxicity.

**Table 2:** The inhibitory effect of natural compounds on RANKL‐induced osteoclastogenesis

Compounds name	Origins	Inhibitory effect on RANKL‐induced osteoclastogenesis
Astilbin	Natural	IC_50_ ≈ 5 μmol/L
Shikonin	Natural	Toxic
Gentiopicroside	Natural	IC_50_ > 10 μmol/L or no effect
Stachydrine hydrochloride	Natural	IC_50_ > 10 μmol/L or no effect
Tubeimoside	Natural	Toxic
Dicoumarol	Natural	IC_50_ > 10 μmol/L or no effect
Echinacoside	Natural	IC_50_ ≈ 7 μmol/L

Note

IC_50_, half maximal inhibitory concentration.

### 3.2 Astilbin attenuates OC resorptive activity

**Figure 3:**
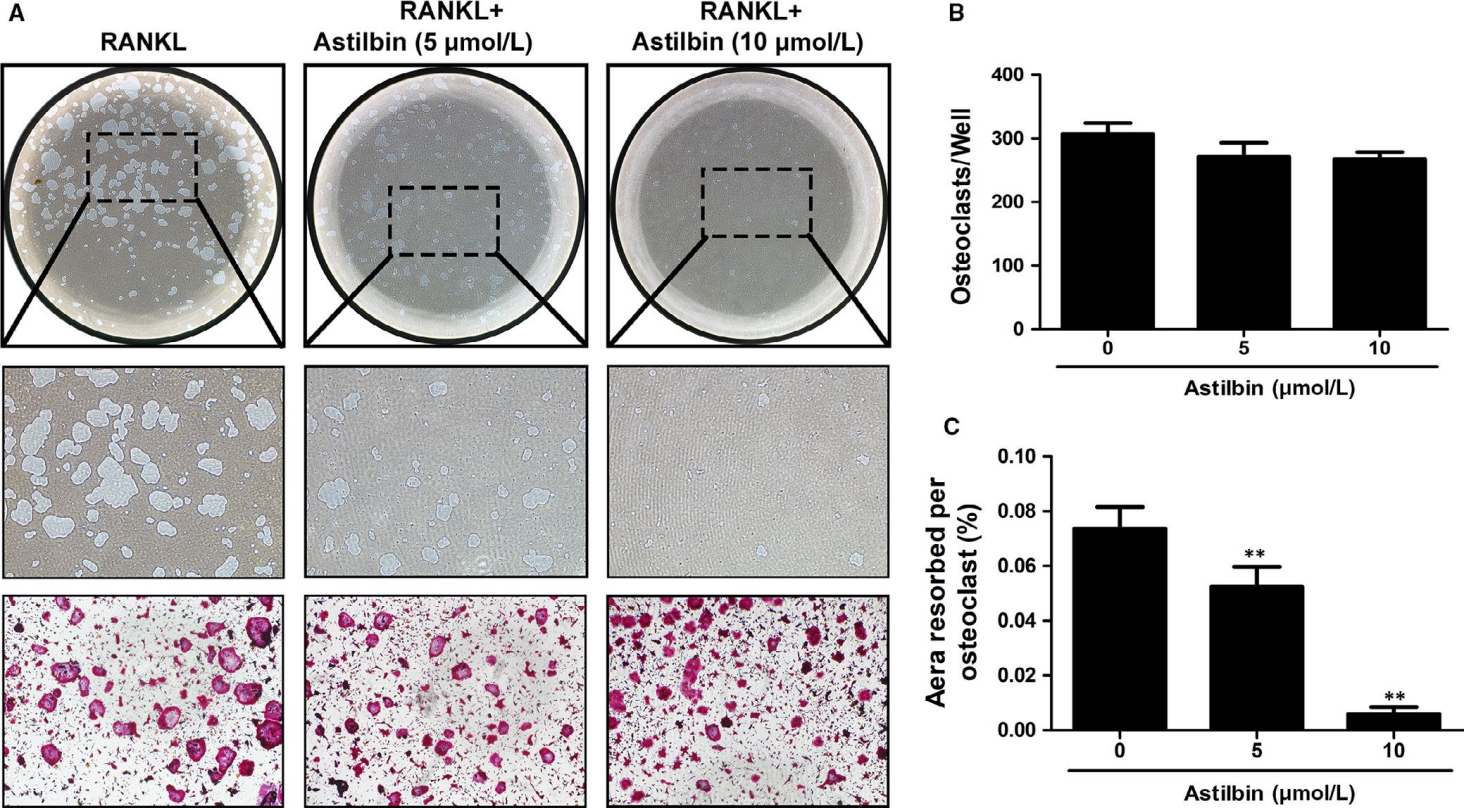
Astilbin suppresses osteoclastic bone resorption activity. A, Representative images of eroded areas and TRAcP‐stained cells on hydroxyapatite‐coated plates in the presence or absence of astilbin (magnification = ×4). B, Quantification of the TRAcP^+^ cells in each well (96‐well plate). C, The resorbed proportion per well after treatment with the indicated concentrations of astilbin was quantified. (**P < .01 relative to the RANKL‐treated control, n = 3)

To investigate whether astilbin can attenuate the cellular resorptive function of OCs, a hydroxyapatite resorption assay was adopted. After treating the cells with varying concentrations of astilbin (0, 5 and 10 μmol/L) for 48 hours, the percentage of resorption areas and the number of OCs per well were measured. As depicted in Figure 3, the number of OCs in each well exhibited no significant differences among the treatment groups, which is consistent with the above‐mentioned conclusion that astilbin inhibits osteoclastogenesis mainly at an early stage. However, the resorption area decreased with increasing drug concentration, especially at 10 μmol/L, which indicates that astilbin can suppress osteoclastic hydroxyapatite resorption activity.

### 3.3 Astilbin inhibits the expression of genes related to RANKL‐induced OC formation and function

**Figure 4:**
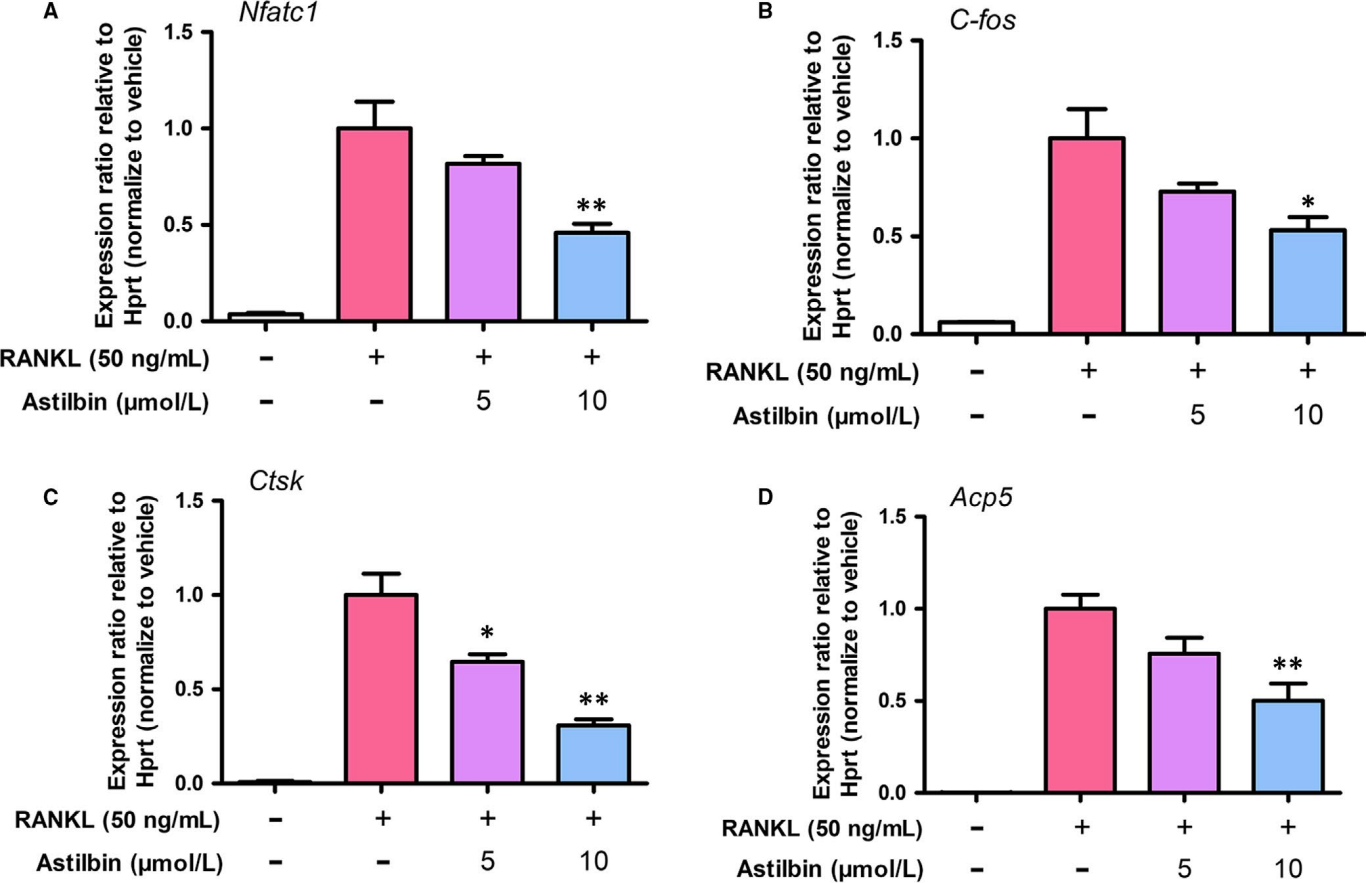
Astilbin blocks osteoclast‐specific gene expression. A, NFATc1, B, C‐Fos, C, CTSK and D, TRAcP (Acp5). Gene expression was standardized to Hprt expression. Data are presented as means ± SD; *P < .05; **P < .01 relative to RANKL‐induced controls

Our results showed that astilbin could suppress OC formation and function. To further explore the underlying mechanism of this effect, real‐time PCR was applied to observe alterations in osteoclast‐related genes. The expression of two key genes (NFATc1 and c‐Fos) during the regulation of OC differentiation was significantly inhibited by astilbin compared with that in the untreated control (Figure 4A, B). Furthermore, the levels of bone resorption‐related genes, including *Acp5* and *CTSK,* were remarkably decreased after astilbin treatment (Figure 4C, D). These observations are consistent with the inhibitory effects of astilbin on osteoclastogenesis and resorption activity, as described above.

### 3.4 Astilbin attenuates NFATc1 activity and downstream protein expression

**Figure 5:**
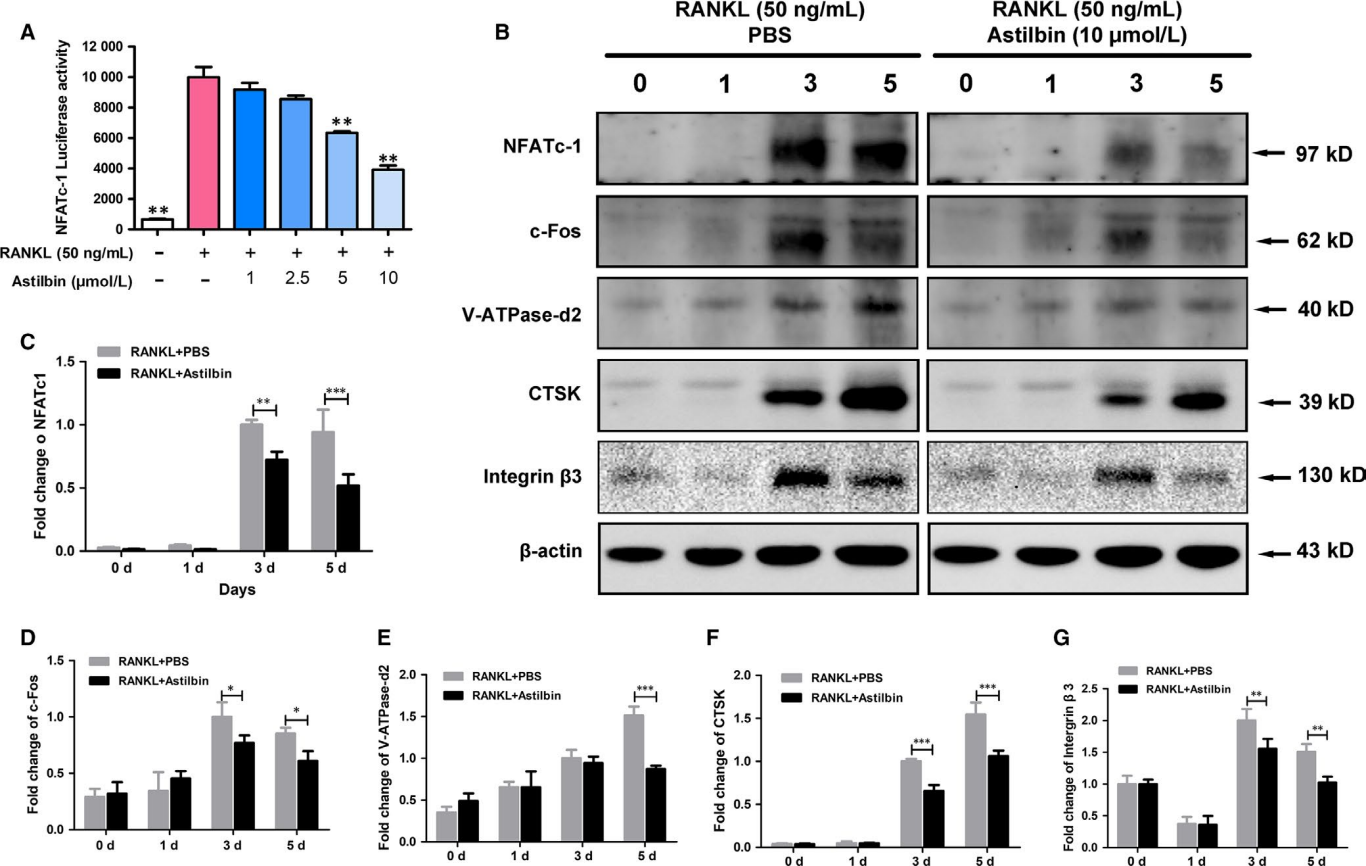
Astilbin represses NFATc1 activity and downstream protein expression. A, The bar graph depicts the NFATc1 luciferase activity of RAW264.7 cells stably transfected with an NFATc1 luciferase reporter construct. Cells were treated with varying concentrations of astilbin and stimulated by GST‐rRANKL (50 ng/mL) for 24 h. B, The protein expression of NFATc1, c‐Fos, V‐ATPase‐d2, CTSK and integrin β3 at day 0, day 1, day 3 and day 5 after stimulation by GST‐rRANKL (50 ng/mL) with or without astilbin (10 µmol/L). C‐G, Analysis of the statistical significance of the difference in protein expression between the astilbin‐treated group and control group. The expression of all proteins mentioned above was determined relative to β‐actin expression. The data in the figures represent means ± SD. Significant differences between the treatment and control groups are indicated as *P < .05, **P < .01 and ***P < .001

To further investigate the mechanism by which astilbin inhibits OC differentiation and activity, a luciferase reporter assay was used to detect the activity of NFATc1. RAW 264.7 cells transfected with NFATc1 reporter construct were treated with varying doses of astilbin. As presented in Figure 5A, the activity of NFATc1 was significantly down‐regulated by astilbin at concentrations of 5 and 10 μmol/L. In addition, astilbin significantly inhibited the expression of the NFATc1 protein, which was remarkably up‐regulated 3 and 5 days after RANKL treatment (Figure 5B, C). Furthermore, the expression levels of downstream proteins related to OC bone resorption activity, such as V‐ATPase‐d2, CTSK and integrin β3, were also decreased by astilbin, mainly on days 3 and 5 (Figure 5B, E‐G). As a component of AP‐1, c‐Fos, a regulator of NFATc1, was also restricted by astilbin at the same time‐points (Figure 5B, D).

### 3.5 Astilbin suppresses RANKL‐induced NF‐κB activation and MAPK phosphorylation during osteoclastogenesis

**Figure 6:**
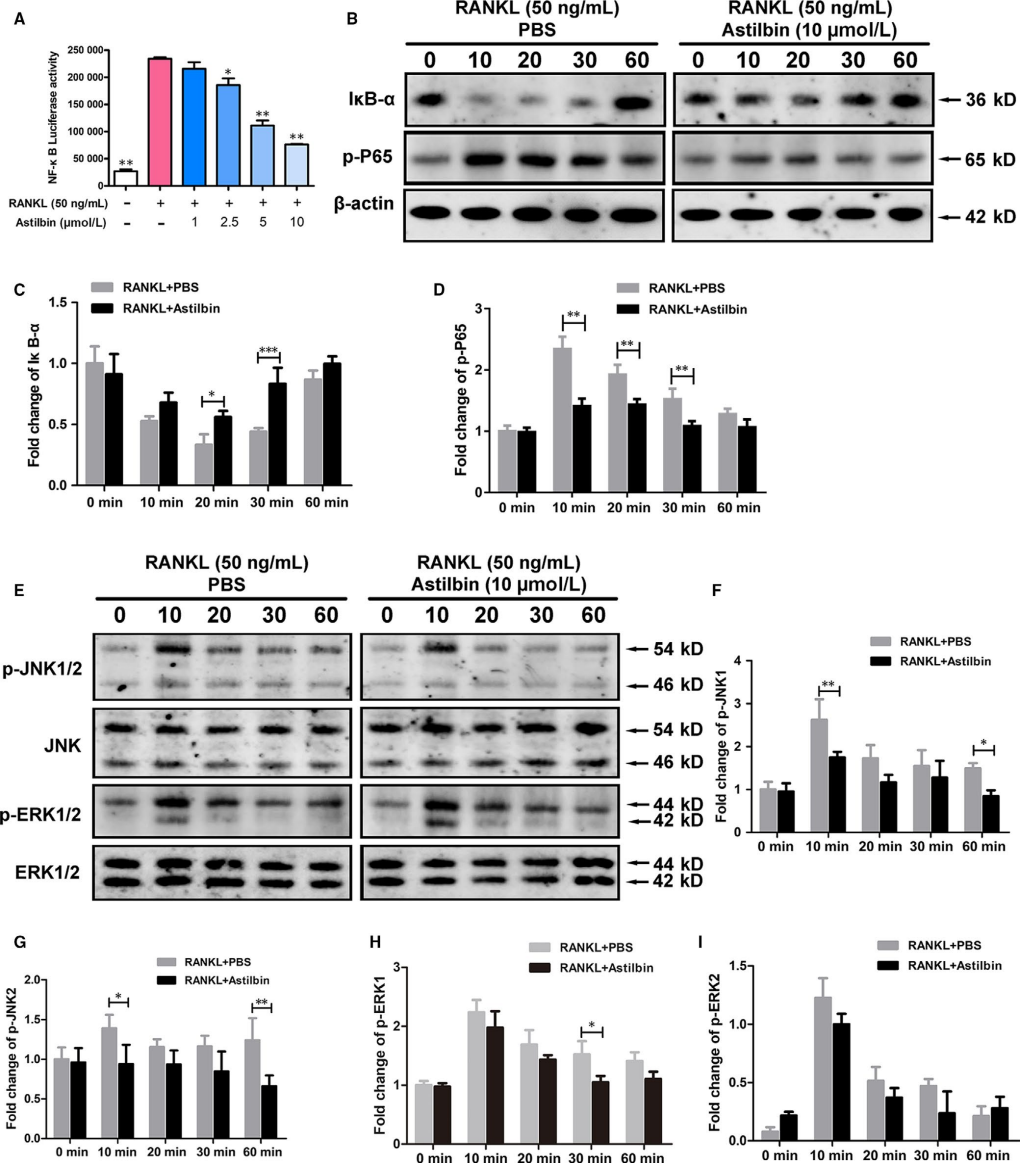
Astilbin suppresses RANKL‐induced NF‐κB activation and MAPK phosphorylation during osteoclastogenesis. A, The bar graph depicts the NF‐κB luciferase activity of RAW264.7 cells stably transfected with an NF‐κB luciferase reporter construct. Cells were treated with varying concentrations of astilbin and stimulated by GST‐rRANKL (50 ng/mL) for 6 h. B, Representative images of Western blots reflecting the expression level of IĸB‐α and p‐P65 normalized to β‐actin. C, D, Quantitative analysis of the fold change in IĸB‐α and p‐P65 expression after astilbin (10 μmol/L) treatment. E, Representative Western blot images of p‐JNK1/2, JNK, p‐ERK1/2, ERK and β‐actin. F‐I, The relative ratio of phosphorylated proteins to unphosphorylated proteins was quantitatively determined. The data in the figures represent means ± SD. Significant differences between the treatment and control groups are indicated as *P < .05, **P < .01, and ***P < .001

The NF‐**κ**B and MAPK signalling pathways play a critical role during OC differentiation and formation. The ability of astilbin to inhibit RANKL‐induced NF‐κB activation was confirmed by an NF‐κB luciferase reporter assay. The results showed that the activation of NF‐κB was suppressed by astilbin in a dose‐dependent manner at concentrations above 2.5 μmol/L (Figure 6A). Inhibitor of NF‐κB (IκB) is a major signalling molecule related to the activation of NF‐κB. Astilbin also inhibited IκB degradation at 20‐30 minutes relative to the degree of inhibition in the control group (Figure 6B, C), which further proved the ability of astilbin to suppress NF‐κB activation during RANKL‐induced osteoclastogenesis. In addition, we used Western blotting to assess the effect of astilbin on the MAPK signalling pathway. The MAPK signalling pathway was represented mainly by ERK, JNK and p38. We found that phosphorylation of JNK, p38 and ERK were inhibited by astilbin during different periods (Figure 6E‐I). Therefore, these data illustrated that astilbin inhibited the formation and function of OCs by inhibiting the NF‐κB and MAPK signalling pathways.

### 3.6 Astilbin decreases ionic calcium oscillations

**Figure 7:**
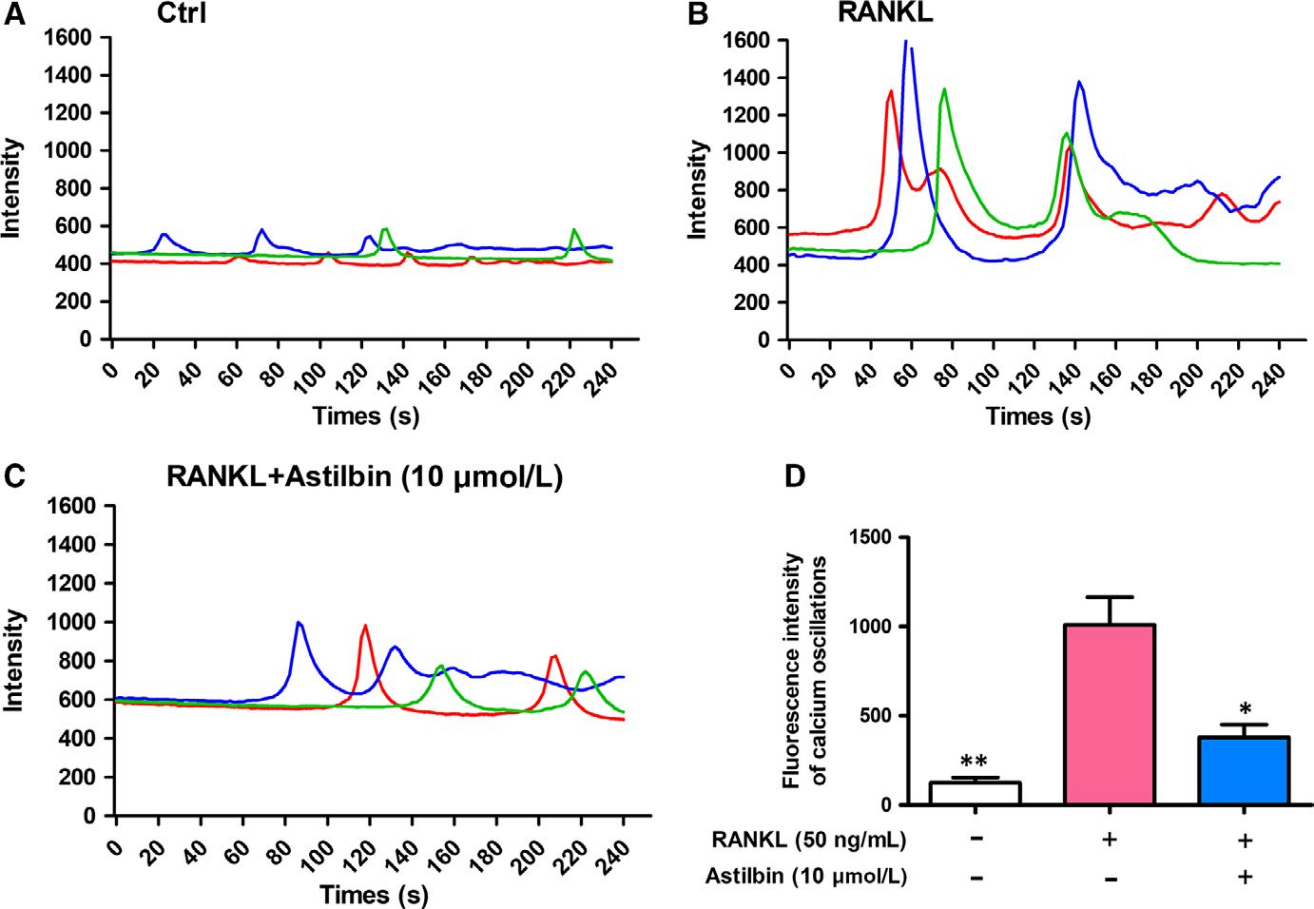
Astilbin disturbs RANKL‐mediated calcium oscillation. A, Representative images of Ca^2+^ oscillation patterns without stimulation by RANKL (M‐CSF only). B, Representative images of the calcium fluctuation pattern stimulated by RANKL. C, Representative images of the calcium fluctuation pattern with astilbin (10 µmol/L) + RANKL. D, Quantitative analysis of the amplitude of the fluorescence intensity of calcium oscillations in each group. Lines of different colours in each image represent the results of three independent experiments. The data in the figures represent means ± SD. Significant differences between the treatment and control groups are indicated as *P < .05, **P < .01, and ***P < .001

Ca^2+^ oscillations, which are initiated by RANKL‐stimulated Ca^2+^ signal transduction pathways, contribute to the activation of NFATc1. Given that astilbin can inhibit NFATc1 activity, we further tested the effects of astilbin on oscillations of ionic calcium in the cytoplasm (Figure 7). As expected, RANKL‐mediated calcium oscillations were decreased by nearly 40% after treatment with astilbin (10 µmol/L). This phenomenon indicated that astilbin impaired RANKL‐mediated calcium oscillations, which is consistent with the suppression of NFATc1 activation, as demonstrated above.

### 3.7 Astilbin ameliorates OVX‐induced systematic bone loss

**Figure 8:**
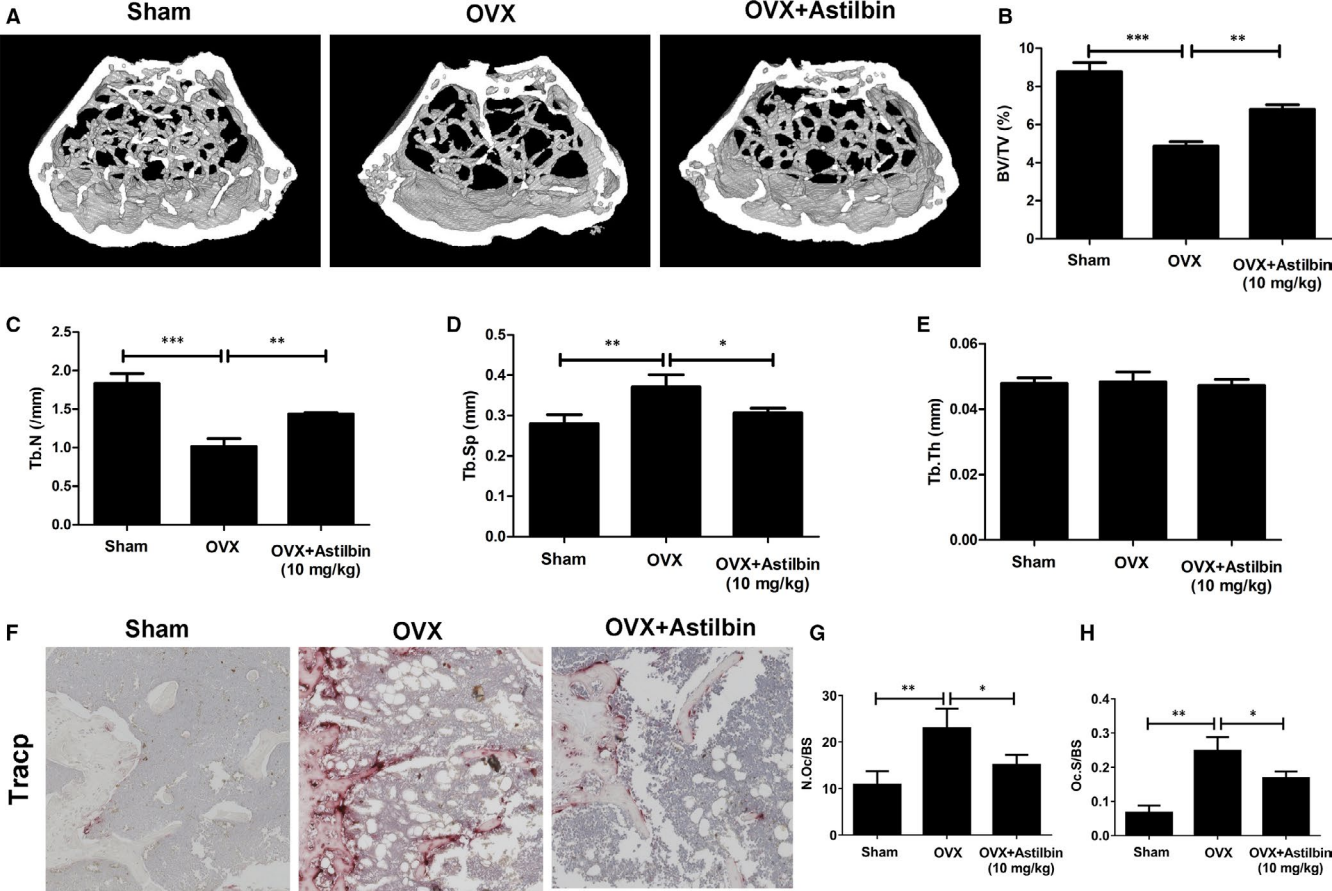
Astilbin ameliorates OVX‐induced systematic bone loss. A, The femur structure captured by high‐resolution µCT was post‐processed by 3D computer reconstruction. B‐E, Quantitative measurements of bone microstructure‐related parameters, such as BV/TV, Tb.N, Tb.Th and Tb.Sp, among the Sham + vehicle, OVX + vehicle and OVX + astilbin (10 mg/kg) groups.(F) Representative images of decalcified bone stained with TRAcP from sham mice, OVX mice and OVX mice treated with 10 mg/kg astilbin. G, H, Quantitative analyses of osteoclast surface/bone surface (Oc.S/BS) and osteoclast number/bone surface (N.Oc/BS). The data in the figures represent means ± SD. Significant differences between the treatment and control groups are indicated as *P < .05; **P < .01 and ***P < .001

In addition to confirming the effect of astilbin on RANKL‐induced osteoclastogenesis at the molecular level, we also conducted animal experiments to evaluate the therapeutic value of astilbin. OVX mice (employed as a model of osteoporotic bone loss) and sham‐operated mice were intraperitoneally injected with astilbin or PBS every other day for 6 weeks. Then, these mice were killed, and their femurs were removed for µCT and quantitative analyses. The results indicated that treatment with astilbin did significantly protect OVX mice from bone loss, with increased bone volume density (BV/TV) and trabecular number (Tb.N) values relative to those of the groups treated with PBS. Meanwhile, trabecular spacing, which was enlarged after OVX, decreased after astilbin treatment. However, the results showed no obvious difference between the groups (Figure 8). Overall, our findings suggest that astilbin has therapeutic value in protecting against systematic bone loss in OVX animals.

## 4 DISCUSSION

Because natural molecules have become promising therapeutic agents for treating osteoporosis by inhibiting RANKL‐induced osteoclastogenesis, it is necessary to study the mechanisms and value of these molecules for therapeutic use.^30^, ^31^ Unlike current treatments, which are challenged by side effects such as osteonecrosis and hormonal disorders,^32^ astilbin demonstrates good application value for the treatment of osteolytic diseases.^12^, ^33^

**Figure 9:**
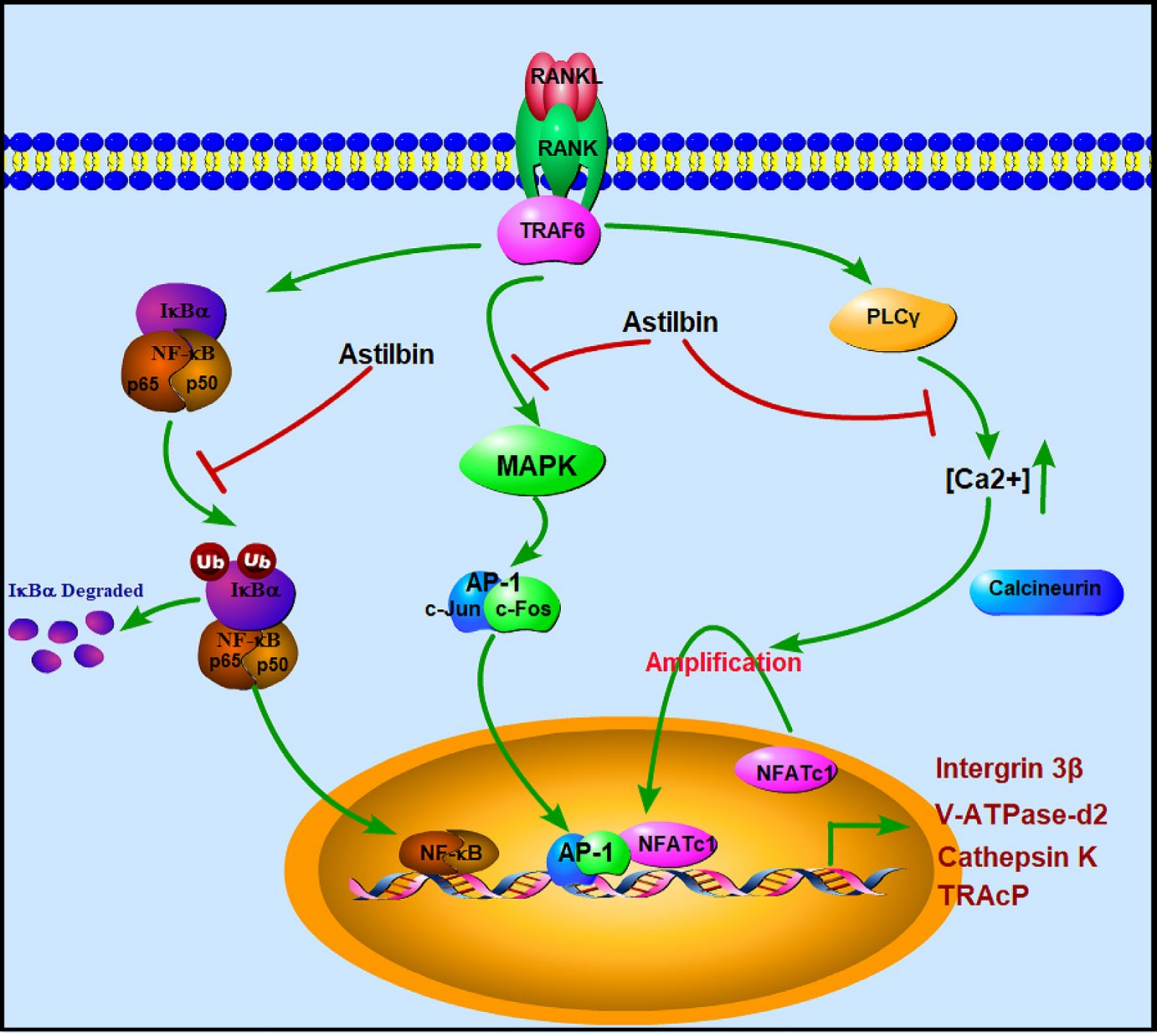
Aschematic diagram for understanding the role of astilbin in suppressing RANKL‐induced osteoclastogenesis

Astilbin, isolated from *Rhizoma Smilacis Glabrae* (a type of Chinese medicinal herb), has been reported to exert numerous bioactivities, including antioxidative and antibacterial activities.^34^ The compound also demonstrates functionality for the treatment of autoimmune diseases.^35^, ^36^ Studies have reported that astilbin exerts pharmacological effects by blocking the NF‐κB signalling pathway and alleviating MAPK signalling cascades.^36^, ^37^ Because these two signalling pathways are also involved in RANKL‐induced osteoclastogenesis, we considered whether astilbin inhibited the differentiation of OCs. In this study, we found that astilbin had a significant inhibitory effect on the RANKL‐induced formation and differentiation of OCs. After treatment with astilbin, both the number and size of OCs were obviously suppressed. The resorption activity of OCs was also inhibited. In addition, the in vivo results further demonstrated the therapeutic value of astilbin in protecting against systematic bone loss. The findings demonstrate that astilbin has great value in ameliorating osteoporotic bone loss (Figure 9).

Bone absorption is an important function of OCs in the body. Once OCs attach to the membrane of the bone surface, a sealing zone will form to promote firmer attachment.^38^ Then, the OCs will secrete numerous enzymes, such as CTSK and TRAcP.^39^ CTSK plays a major role in degrading the bone matrix, while TRAcP mainly enhances the activity of CTSK.^40^, ^41^ In our study, the expression levels of CTSK, Acp5 (TRAcP) V‐ATPase‐d2 and integrin β3 were significantly inhibited after treatment with astilbin, which was related to the down‐regulation of RANKL‐induced osteoclastic bone resorption. The bone resorption‐related proteins mentioned above are the downstream proteins stimulated by NFATc1.^42^ NFATc1 is an indispensable transcription factor that participates in the formation of OCs from BMMs.^43^ A previous study found that the autoamplification of NFATc1 was mediated by Ca^2+^ oscillation induced by RANKL, which demonstrated that NFATc1 was regulated by the Ca/calcineurin pathway.^44^ NFATc1 was also regulated by c‐Fos. This finding was confirmed by a study that found that the expression of RANKL‐induced NFATc1 was abrogated in c‐Fos knockout mice.^45^ In addition, another study demonstrated that mice developed osteopetrosis because of c‐Fos deficiency, which also demonstrated the importance of c‐Fos in RANKL‐induced osteoclastogenesis.^46^ In our study, the activities of NFATc1, c‐Fos and Ca^2+^ oscillation were all inhibited by astilbin. These results indicate that astilbin exerts a strong inhibitory effect on the formation and function of OCs by inhibiting NFATc1 activity.

NF‐κB acts as an initiator of NFATc1 induction during RANKL‐induced osteoclastogenesis. It was reported that the activity of NFATc1 was significantly suppressed after treatment with dehydroxymethylepoxyquinomicin (DHMEQ) (an NF‐κB inhibitor).^5^ Another experiment also demonstrated that p50 and p65 (two components of NF‐κB) activated the NFATc1 promoter 1 hour after RANKL interacted with RANK, which illustrates the close relationship between NF‐κB and NFATc1.^47^, ^48^ In the cytoplasm of non‐stimulated cells, NF‐κB exists with IκB as a complex. IκB is then degraded by inhibitor of κB kinase (IKK) after RANKL stimulation, whereas NF‐κB enters the nucleus and stimulates the transcription of key genes.^49^ Therefore, IκB can be regarded as a signalling molecule that reflects NF‐κB activation. As shown in our study, both the activation of NF‐κB and the degradation of IκB were repressed by astilbin. Therefore, the positive effect of astilbin on the prevention of OC formation may be caused by the inhibition of NF‐κB.

AP‐1, another major transcription factor in OC differentiation, was also found to be inhibited by astilbin. C‐Fos and c‐Jun are the components that form AP‐1. MAPK, which consists of JNK, ERK and p38, is a part of the RANKL‐induced signalling pathway that regulates the expression of AP‐1,^50^ which means the phosphorylation of components in the MAPK signalling pathway also regulates the transcription of c‐Jun and c‐Fos.^25^, ^51^ In addition, it has been reported that ERK contributes to the protection of OCs from apoptosis and the stimulation of OC differentiation.^52^ JNK blockade also leads to the failure of OC formation. Although p38 is not involved in OC function, this protein does participate in OC differentiation.^53^, ^54^ In our study, the phosphorylation of components in the MAPK signalling pathway was inhibited by astilbin, which explained the AP‐1 suppression described above and indicated that astilbin repressed OC formation by reducing the phosphorylation of components of the MAPK signalling pathway.

Unlike osteoclasts, osteoblasts are responsible for bone formation and also have a main role in the mineralization of bone structures. Therefore, the effect of astilbin on osteoblasts was also analysed in our study. However, no significant difference was found between the astilbin and control groups. In addition, the result of the MTS assay showed that astilbin was not toxic to osteoblasts at concentrations of 20 μmol/L and lower. Collectively, astilbin had no effect on the differentiation and mineralization of osteoblasts.

In addition to the above‐mentioned evidence for the inhibitory effect of astilbin at the cellular level, our results obtained from the OVX mouse model showed that astilbin could improve bone quality from bone loss, which further confirmed its promising role in future treatment.

In conclusion, our study indicates that astilbin can inhibit RANKL‐induced osteoclastogenesis by targeting NFATc1, NF‐κB and MAPK signalling and preventing bone loss in OVX mice. This study provides promising results to the prevention of osteolytic diseases and a mechanistic basis for the development and utilization of traditional Chinese medicine for skeletal conditions.^30^, ^31^

## CONFLICT OF INTEREST

The authors declare no conflicts of interest.

## AUTHOR'S CONTRIBUTION

Jiake Xu, Haiming Jin, Qingqing Wang and Kai Chen performed the experiments and metagenomics analysis and drafted the manuscript. Ke Xu, Hao Pan and Feifan performed the animal experiment. Zhen Ye, Ziyi Wang and Jennifer Tickner performed the histological and histomorphometric analysis. Heng Qiu, Chao Wang and Jacob Kenny helped to do the cell experiment. Huazi Xu and Te Wang helped to design the study and revise the manuscript.
