# Supplementary figures and images for: Astilbin prevents bone loss in ovariectomized mice through the inhibition of RANKL‐induced osteoclastogenesis
Source: J Cell Mol Med. 2019 Oct 11;23(12):8355–68. doi: 10.1111/jcmm.14713 (PMC6850941; doi:10.1111/jcmm.14713)

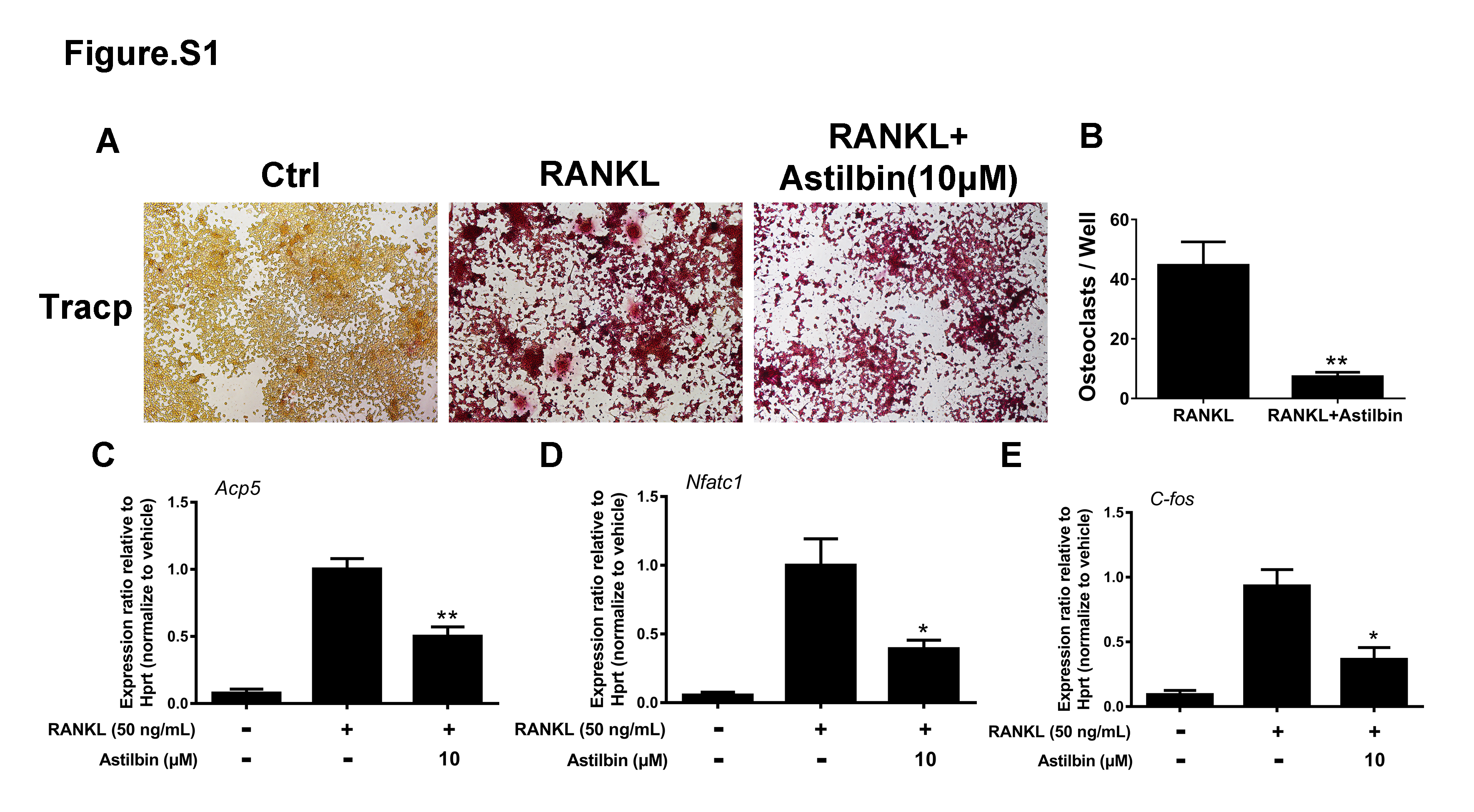

Supplement: Supplementary file 1 [file JCMM-23-8355-s001.tif]

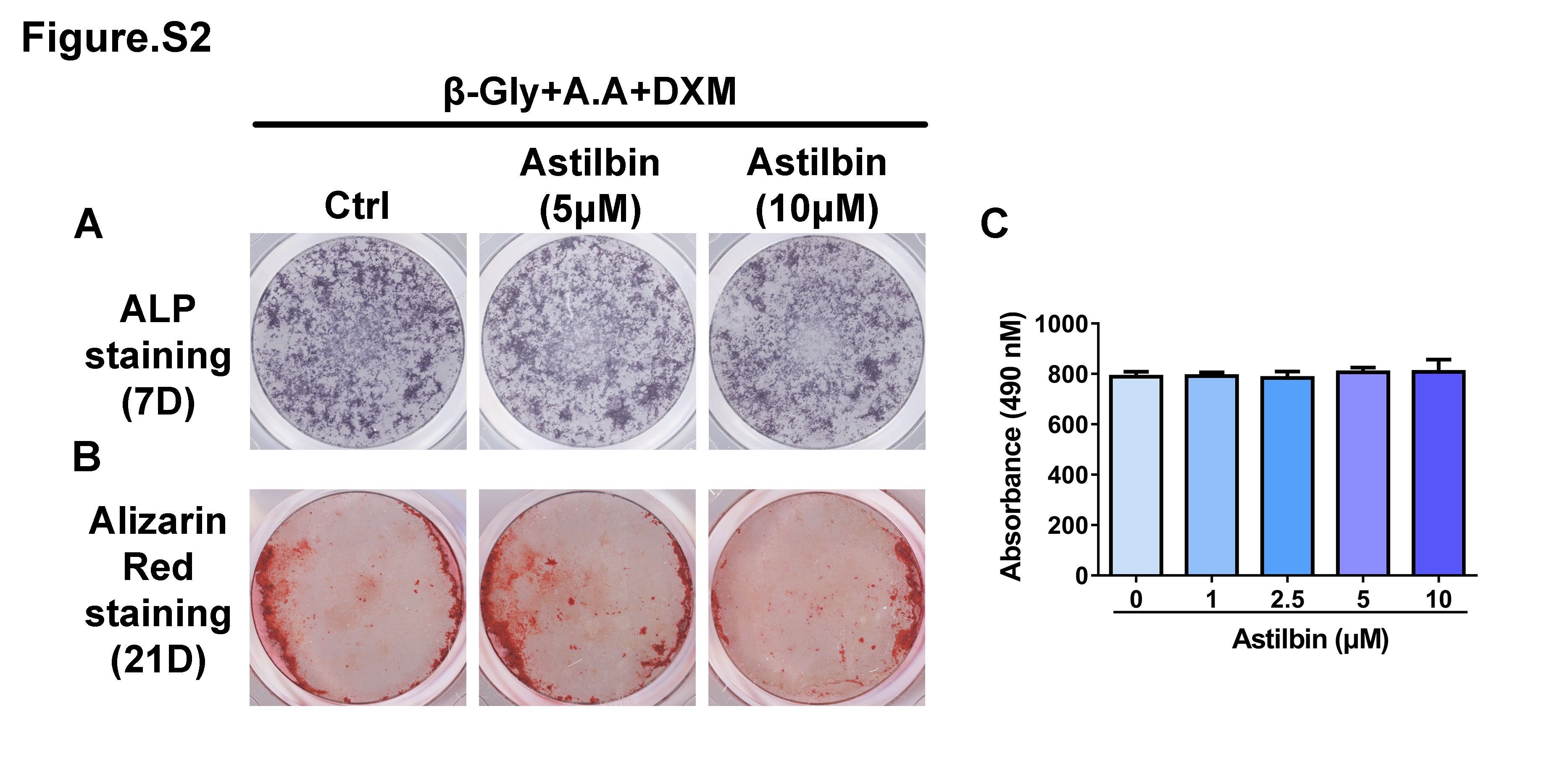

Supplement: Supplementary file 2 [file JCMM-23-8355-s002.tif]
